# Gut microbiota–derived polyamine pathways associated with mean blood pressure

**DOI:** 10.1038/s41440-025-02490-8

**Published:** 2025-12-18

**Authors:** Yasuo Ikagawa, Shigefumi Okamoto, Kouki Taniguchi, Ren Mizoguchi, Atsushi Hashimoto, Rikako Imamura, Hiroshi Arakawa, Kohei Ogura, Masashi Yanagihara, Hiromasa Tsujiguchi, Akinori Hara, Hiroyuki Nakamura, Kazuyoshi Hosomichi, Shigehiro Karashima

**Affiliations:** 1 Laboratory of Medical Microbiology and Microbiome, Department of Clinical Laboratory and Biomedical Sciences, Division of Health Sciences, Graduate School of Medicine, The University of Osaka, Osaka, Japan; 2 Department of Clinical Laboratory Science, Faculty of Health Sciences, Institute of Medical, Pharmaceutical and Health Sciences, Kanazawa University, Kanazawa, Japan; 3 Department of Health Promotion and Medicine of the Future, Kanazawa University, Kanazawa, Japan; 4 Faculty of Pharmacy, Institute of Medical, Pharmaceutical and Health Sciences, Kanazawa University, Kanazawa, Japan; 5 Department of Regulatory Science, Graduate School of Pharmaceutical Sciences, Nagoya City University, Nagoya, Japan; 6 Laboratory of Basic and Applied Molecular Biotechnology, Division of Food Science and Biotechnology, Graduate School of Agriculture, Kyoto University, Uji, Japan; 7 Department of Public Health, Graduate School of Advanced Preventive Medical Sciences, Kanazawa University, Kanazawa, Japan; 8 Laboratory of Computational Genomics, School of Life Science, Tokyo University of Pharmacy and Life Sciences, Hachioji, Japan; 9 Institute of Liberal Arts and Science, Kanazawa University, Kanazawa, Japan

**Keywords:** Gut microbiome, Polyamines, Spermidine, Arginine

## Abstract

Hypertension is a common lifestyle-related disease and is influenced by various factors, including excessive salt intake. Recently, the gut microbiota (GM) has gained attention for its potential involvement in blood pressure regulation; however, polyamine metabolism involvement remains poorly understood. Sixty participants aged ≥40 years from Shika Town, Japan, were stratified into four groups (*n* = 15 each) based on mean blood pressure and urinary sodium chloride (u-NaCl) excretion. The clinical parameters were evaluated, and fecal samples were analyzed using shotgun metagenomic sequencing to assess the microbial composition and abundance of genes related to arginine–polyamine metabolism. Three major findings were observed: (1) Significant differences in the α-diversity of GM were observed between salt-sensitive and non–salt-sensitive hypertensive groups; (2) The abundance of spermidine synthase (EC 2.5.1.16), a key enzyme in polyamine metabolism with known antihypertensive effects, was significantly higher in normotensive individuals, independent of u-NaCl excretion; and (3) Bacterial species harboring polyamine metabolic enzyme genes, including EC 2.5.1.16, differed significantly between groups, suggesting group-specific microbial metabolic traits. These findings suggest that GM-mediated polyamine metabolism may contribute to the regulation of salt-sensitive blood pressure. While variations in spermidine-producing bacteria and the involvement of EC 2.5.1.16 were observed, these factors alone do not fully account for the intergroup differences related to salt intake. Thus, polyamine metabolism likely plays a part in salt sensitivity, but additional microbial and host factors are also involved. Further studies are needed to validate these findings and to explore microbiota-targeted strategies for the prevention and treatment of hypertension.

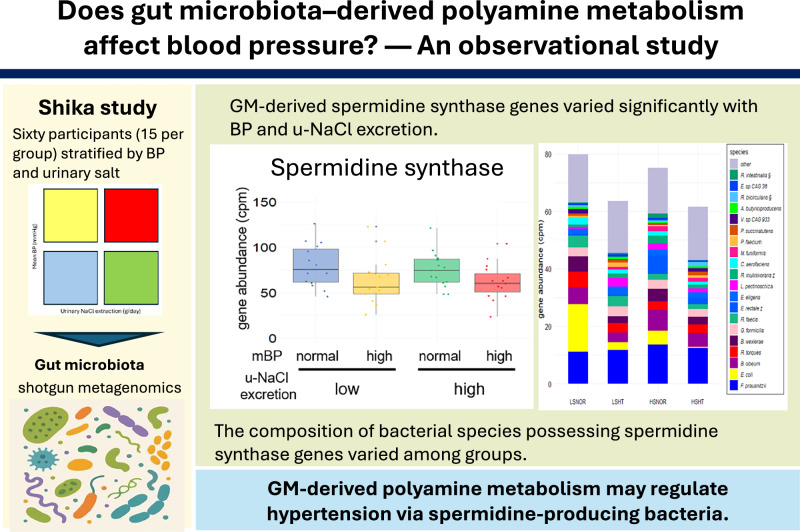

## Introduction

Hypertension (HT) is a major risk factor for cardiovascular disease [1]. The World Health Organization reports that an estimated 30% of adults suffer from HT, which is responsible for several serious health problems, including myocardial infarction, stroke, and renal failure [2]. Early detection and proper management of HT can significantly reduce the risk of complications. Therefore, understanding the pathophysiology of HT and developing preventive and treatment strategies are important for public health.

HT is caused by a combination of genetic and environmental factors, including excessive salt intake. Studies have shown that excessive sodium intake increases blood pressure (BP) and the risk of cardiovascular diseases [3]. However, none of the participants experienced a similar increase in BP in response to the salt intake. In other words, the effect of salt sensitivity on individual BPs varies [4–6]. Studies have focused on the role of vasodilators, such as nitric oxide (NO) [7–9] and polyamine metabolism as factors and mechanisms of salt sensitivity.

Polyamines are hydrocarbon compounds with two or more amino groups in their molecular structure. Typical polyamines include putrescine, spermidine, and spermine [10]. Spermidine is one of the most studied polyamines and a BP suppressor. Spermidine increases the bioavailability of arginine and is thought to exert antihypertensive effects by enhancing NO production [10]. Spermidine has also been associated with the suppression of inflammation-induced cytokines in animal models of HT, inhibition of oxidative damage to vascular endothelial cells, and improvement in renal dysfunction [10]. Spermidine is synthesized in host cells or ingested from the diet, but is also produced by the intestinal microflora and taken up by the human body [11]. In recent years, there have been several reports supporting the role of GM in the regulation of BP [12–15]; however, no studies have reported on the impact of gut microbiota-derived polyamine metabolism on BP.

In this study, we investigated whether gut microbial polyamine metabolism is associated with BP regulation and how dietary salt intake may influence the abundance of bacteria harboring polyamine metabolism-related genes. This study aims to elucidate the potential role of gut microbiota-derived polyamine metabolism in BP regulation and to provide insights that may inform future preventive and therapeutic strategies.

## Methods

### Study design

This observational study was conducted using data from residents aged 40 years and older who participated in the “Shika Town Super Preventive Health Checkup” conducted in 2017 in Shika Town, Hakui District, Ishikawa Prefecture, Japan. Serum, urine, and fecal samples collected during the health checkup were used to analyze the GM and related metabolites [16].

### Ethical considerations

This study was conducted in accordance with the Declaration of Helsinki and approved by the Ethics Committee of the Graduate School of Medical Sciences, Kanazawa University (Approval No. 1491). Written informed consent was obtained from all participants after receiving a detailed explanation of the study objectives and procedures.

### Data collection

Shika-machi Super Preventive Health Checkup data pertaining to parameters such as age, sex, medical history, medication status, and smoking status were collected using a questionnaire. The body mass index (BMI) was calculated by dividing the current weight (kg) by the square of height (m^2^). Participants rested in a chair in the examination room for more than 5 min before BP was measured, and the mean of two stable values was taken as the clinical BP value. BP was measured in a sitting position with an appropriately sized cuff placed on the right upper arm. The sphygmomanometers used were UM-15P (Parama-tech Co., Ltd., Fukuoka, Japan) and HEM-907 (OMRON Co., Ltd., Kyoto, Japan), which are digital automatic sphygmomanometers based on the oscillometric method [16]. Serum samples were collected in the morning after fasting for 12 h and resting for 15 min. Urine samples were collected as required. The serum and urine samples were refrigerated immediately after collection. Urinary sodium excretion was assessed using a single morning spot urine sample collected under fasting conditions on the same day as blood sampling during the health check-up. A 24-h urine collection was not performed. The concentration of NO metabolites in the urine (NO/gCr) was measured using the total NO and nitrate/nitrite assay (R&D Systems, Inc., MN, USA). This kit measures the absorbance of nitrate and nitrite in urine, and the total value is considered the urinary NO concentration. The values were adjusted using urinary creatinine levels to obtain NO/gCr.

### Selection of participants for analysis

Data from 254 participants who voluntarily submitted stool samples were analyzed out of 460 individuals who underwent health check-ups. The exclusion criteria were as follows: (1) individuals taking BP-lowering medications, steroids, bowel regulators, antibacterial agents, or Proton Pump Inhibitors; (2) patients undergoing cancer treatment; and (3) individuals with missing data. Eighty-nine patients were excluded.

**Fig. 1:**
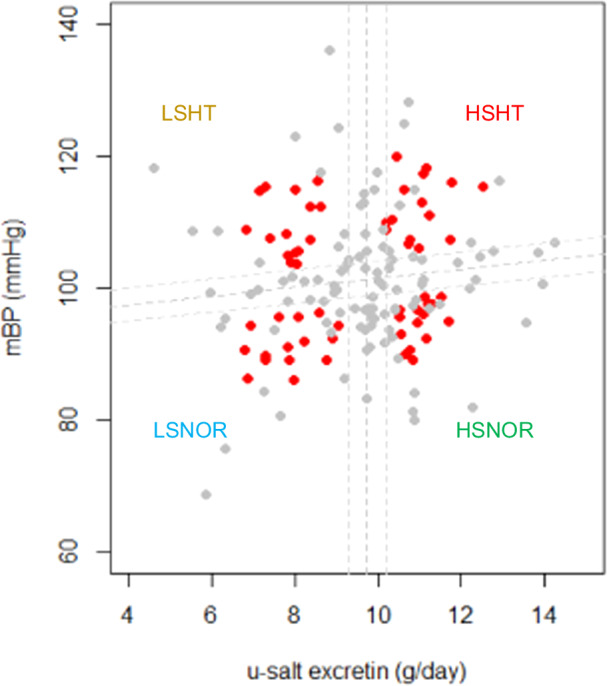
Selection of participants for analysis based on mean blood pressure and urinary sodium chloride excretion. A total of 165 participants were evaluated based on mean blood pressure (mBP) and urinary sodium chloride (u-NaCl) excretion. The vertical axis represents mBP, while the horizontal axis represents the u-NaCl excretion. The dashed lines in the center of the scatter plot indicate the regression line for mBP and the median value of u-NaCl excretion. The median u-NaCl excretion was determined to be 9.7 g/day. Dashed lines at both ends of the plot represent the range within ±0.25 standard deviations (SD) from the median. Participants who were within ±0.25 SD from both the regression line and the median were excluded from further analysis. Subsequently, k-means clustering was used to identify the centroids of four distinct clusters. From these clusters, the 15 cases with the shortest Euclidean distance from each centroid were selected for analysis. The red dots in the plot indicate the participants chosen for this analysis. The gray dots indicate participants who were not selected

Scatter plots of the mean BP (mmHg) and urinary sodium chloride (u-NaCl) excretion (g/day) for the 165 participants are shown (Fig. 1). The participants were classified into four categories using a scatter plot regression line and median u-NaCl excretion (9.7 g/day). Participants located within ±0.25 standard deviations from both the regression and median lines were omitted to mitigate potential outliers. Subsequently, the centroid of each cluster was determined using the k-means method. From each of the four delineated clusters, 15 samples with the shortest Euclidean distances from their respective centroids were selected for subsequent analyses. Participants were categorized into four groups: low-salt/normal BP (LSNOR), low-salt/high BP (LSHT), high-salt/normal BP (HSNOR), and high-salt/high BP (HSHT).

### Fecal microbiota shotgun metagenomic analysis

Fecal samples were collected and stored as previously described [16]. The fecal samples were processed in a Biosafety Level 2 laboratory. The total DNA was extracted from fecal samples using the NucleoSpin® DNA Stool (Macherey-Nagel Inc., Düren, Germany). Shotgun metagenomic analysis of the fecal samples was performed by GeneBay, Inc. DNA was fragmented using a Covaris ME220 (Covaris Inc., Woburn, MA, USA), purified, and size-sectioned using AMPure XP beads (Beckman Coulter Inc., Brea, CA, USA). Processed samples were prepared using the MGIEasy PCR-Free DNA Library Prep Set (MGI Tech Co., Ltd., Shenzhen, China), and libraries with index barcodes were prepared. The resulting library was sequenced (2 × 150 bp) using a next-generation sequencer, DNBSEQ-G400 (MGI Tech Co., Ltd.), to obtain raw read data (FASTQ format).

### Management of sequence data

Raw read data obtained by shotgun metagenomic sequencing were quality-checked using FASTP (version 0.20.1) [17]. Low-quality reads were removed using Trimomatic software (version 0.33) [18]. Human-derived genomic sequences were removed using a two-step process. First, all reads were aligned against the human reference genome (hg37dec_v0.1) using Bowtie2 (version 1.1.2) [19] to identify and remove human-derived sequences. KneadData (version 0.10.0; Huttenhower Lab, Harvard University) was used to purge remaining human-derived reads from the dataset. Sequences that were not removed were retained for the microbiome analysis.

### Microbiome analysis

A bacterial flora composition analysis was performed using the default settings of the Metagenomic Phylogenetic Analysis tool v.3.0 (MetaPhlAn3) [20], and the relative abundance ratios of the bacterial species were calculated. The mpa_v30_CHOCOPhlAn_201901 database was used to map bacterial species.

The standard settings of the Human Microbiome Project Unified Metabolic Analysis Network (HUMAnN3, version 3.0.10) [21] were used for functional analysis of the bacterial flora, and the abundance of enzyme genes and metabolic pathways from bacterial sources was calculated. As the first step in this process, the bacterial genomes identified by MetaPhlAn3 were mapped to the pan-genome database chocophlan.v296_201901b using Bowtie2 (version 1.1.2) [19]. As the first step in this process, the bacterial genomes identified by MetaPhlAn3 were mapped to the pan-genome database ChocoPhlAn (version chocophlan.v296_201901b) using Bowtie2 (version 1.1.2) [19]. Reads that could not be mapped to ChocoPhlAn—representing sequences of unknown bacterial origin—were subsequently aligned to the UniRef90 [22] protein database (uniref90_annotated_v201901b) using DIAMOND (version 2.0.9) [23]. Finally, enzyme gene abundances were mapped to metabolic pathways using the MetaCyc database (mapping_v201901b) [24].

### Enzyme genes related to polyamine metabolism

Enzyme genes related to polyamine metabolism were identified by querying the MetaCyc database [24], in order to extract candidate genes for downstream screening in our dataset. The targeted metabolic pathways are shown in Supplementary Fig. S1. We examined pathways converting arginine to spermidine via agmatine and putrescine, as well as to ornithine and citrulline. In this study, only genes that could be functionally annotated in existing reference databases (ChocoPhlAn, UniRef90, and MetaCyc) were included in the analysis, and unannotated or unknown genes were excluded. Therefore, the results represent known arginine- and polyamine-related enzyme genes currently registered in these databases.

### Statistical analysis

All statistical analyses were performed using R-studio software (version 4.3.1; Boston, MA, United States). Group comparisons were performed between the two groups with low u-NaCl excretion (LSNOR vs. LSHT), two groups with high u-NaCl excretion (HSNOR vs. HSHT), two groups with normal BP (LSNOR vs. HSNOR), and two groups with high BP (LSHT vs. HSHT). Welch’s t-test was used for significance tests of clinical information, α-diversity, bacterial species, and metabolic enzyme genes. Principal component analysis (PCA) was performed using the Simpson index for α-diversity and the Bray–Curtis distance for β-diversity using R’s “vegan” Package. PERMANOVA was used for significance tests of β-diversity. Analysis of covariance was used to compare metabolite ratios. In accordance with previous studies [16], we selected age, sex, and BMI as possible confounders affecting the GM. For all results of the significant difference tests, a difference was considered statistically significant at *p* < 0.05.

## Results

### Clinical background

Table 1 presents the clinical characteristics of the 60 patients who underwent shotgun sequencing. Both systolic and diastolic BP values differed significantly between the LSNOR and LSHT groups as well as between the HSNOR and HSHT groups (all *p* < 0.01). In addition, the diastolic BP was significantly lower in the LSNOR group than in the HSNOR group (*p* < 0.01). When applying the standard hypertension criteria (systolic BP ≥ 140 mmHg or diastolic BP ≥ 90 mmHg), nine participants showed discrepancies between the classification based on mBP and the conventional hypertension definition. Urinary NaCl excretion significantly differed between the LSNOR and HSNOR groups and between the LSHT and HSHT groups (both *p* < 0.01). No significant differences were observed in other clinical parameters, medical histories, or NO/gCr levels.

**Table 1:** Clinical characteristics of participants

	LSNOR (*n* = 15)	LSHT (*n* = 15)	HSNOR (*n* = 15)	HSHT (*n* = 15)
female, n: %	10 (66.7)	7 (46.7)	6 (40.0)	7 (46.7)
age, y	66 ± 12	63 ± 11	63 ± 7	63 ± 11
BMI, kg/m^2^	21.8 ± 3.2	24.3 ± 3.7	23.5 ± 2.4	24.5 ± 3.2
SBP, mmHg	133.6 ± 12.5	152.3 ± 11.4*	132.2 ± 9.4	154.0 ± 10.0†
DBP, mmHg	70.3 ± 4.0	88.1 ± 5.4*	76.1 ± 4.5‡	91.3 ± 7.2†
u-NaCl excretion, g/day	7.9 ± 0.7	7.9 ± 0.5	11.0 ± 0.3‡	11.0 ± 0.6§
PRA, ng/ml/hr	2.4 ± 2.7	2.1 ± 1.3	1.7 ± 1.6	1.3 ± 0.6
PAC, pg/ml	128.8 ± 33.4	155.1 ± 62.3	124.1 ± 38.3	124.0 ± 71.2§
NO/gCr, mmol/gCr	0.7 ± 0.3	1.1 ± 0.9	1.6 ± 1.3	1.1 ± 0.8
Current smoking, n: %	2 (13.3)	2 (13.3)	1 (6.7)	1 (6.7)
medical history				
diabetes, n: %	3 (20.0)	2 (13.3)	1 (6.7)	0 (0)
dyslipidemia, n: %	4 (26.7)	4 (26.7)	3 (20.0)	2 (13.3)
IHD, n: %	1 (6.7)	0 (0)	1 (6.7)	1 (6.7)
stroke, n: %	0 (0)	0 (0)	0 (0)	0 (0)
CKD, n: %	0 (0)	0 (0)	0 (0)	0 (0)

Data are presented as mean ± SD or number of subjects (%). The *P*values were calculated by Welch’s t-test. *: *p* < 0.05, LSNOR versus LSHT. †: *p* < 0.05, HSNOR versus HSHT. ‡: *p* < 0.05, LSNOR versus HSNOR. §: *p* < 0.05, HSHT versus LSHT. NO/gCr was measured in LSNOR (*n* = 14), LSHT (*n* = 15), HSNOR (*n* = 14), and HSHT (*n* = 14)

*BMI* body mass index, *SBP* systolic blood pressure, *DBP* diastolic blood pressure, *u-NaCl excretion* urinary- NaCl excretion, *PRA* plasma renin activity, *PAC* plasma aldosterone concentration, *NO/gCr* urinary nitric oxide metabolites/ grams creatinine, *IHD* ischemic heart disease, *CKD* chronic kidney disease

### Diversity of bacterial species

**Fig. 2:**
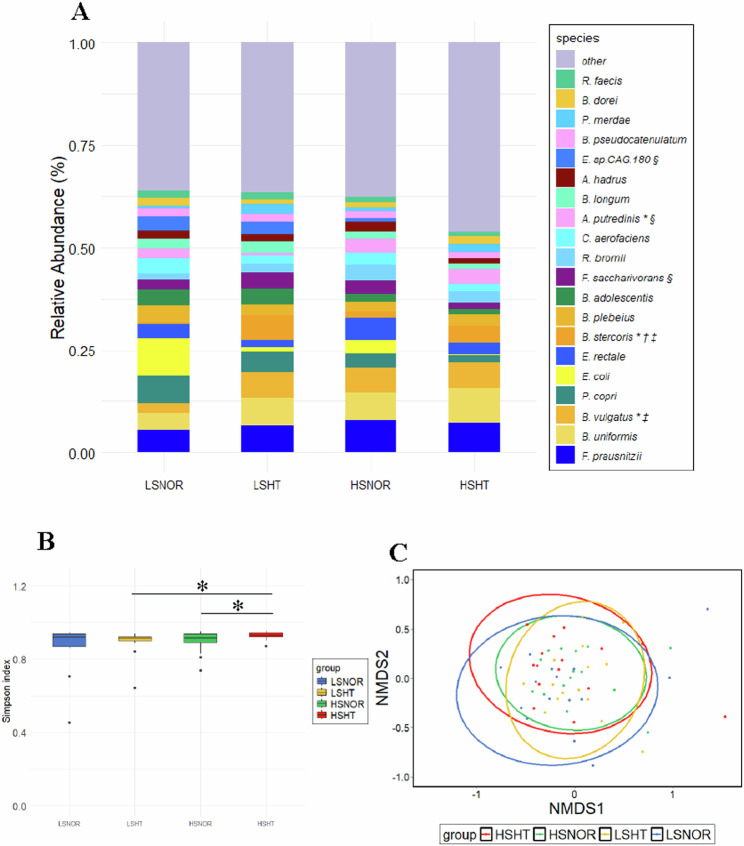
Comparison of GM diversity in four groups according to mBP and salt excretion. The species-level composition of bacterial flora is illustrated in a stacked graph (**A**). The graph depicts the top 20 bacterial species in terms of abundance across all samples, with the remaining species grouped under “Other.” Significant differences between the LSNOR and other groups (*p* < 0.05) are indicated with *p < 0.05, LSNOR vs. LSHT. †: *p* < 0.05, HSNOR vs. HSHT. ‡: *p* < 0.05, LSNOR vs. HSNOR. §: *p* < 0.05, HSHT vs. LSHT. The α-diversity (**B**) and β-diversity (**C**) of the bacterial flora in each group were compared. Simpson’s index was used to evaluate the α-diversity, while PERMANOVA was employed for β-diversity. Welch’s t-test was used to determine the significance of the differences. No significant differences in α-diversity and β-diversity were found between the groups

Figure 2A shows a stacked graph illustrating the average relative abundance ratios of the top 20 bacterial species. The diversity of the intestinal microbiota was assessed across four groups categorized by the mean BP and salt excretion. *Bacteroides vulgatus* (*B. vulgatus*) and *Bacteroides stercoris* (*B. stercoris*) were significantly more abundant in the LSHT group than in the LSNOR group (*p* = 0.010 and 0.006, respectively), while *Alistipes putredinis* (*A. putredinis*) was more abundant in the LSNOR group (*p* = 0.029). *B. stercoris* was significantly more abundant in the HSHT group than in the HSNOR group (*p* = 0.026). *B. vulgatus* and *B. stercoris* were significantly more abundant in the HSNOR group than in the LSNOR group (*p* = 0.007 and 0.026, respectively).*Fusicatenibacter saccharivorans* and *E. sp*. CAG.180 were significantly more abundant in the LSHT group than in the HSHT group (*p* = 0.025 and 0.046, respectively), while *A. putredinis* was more abundant in the HSHT group (p = 0.004).α-Diversity was significantly higher in the HSHT group compared to both the HSNOR and LSHT groups (*p* = 0.041 and 0.043, respectively; Fig. 2B), while no significant differences were found in β-diversity (Fig. 2C).

### Comparison of arginine- and polyamine-related enzyme gene abundance

Supplementary Figure S1A shows a metabolic pathway diagram of the arginine metabolism-related enzyme genes identified in the analyzed fecal samples. Supplementary Figure S1B shows the proportion of bacterial species harboring each enzyme gene among the 60 participants analyzed. Notably, EC 2.1.3.3 and EC 4.1.1.19 were detected in 60.2% and 59.5%, respectively, of the bacterial species isolated from patients. In contrast, the prevalence of bacterial species possessing EC 3.5.1.53 and EC 4.1.1.17 was less than 10%.

**Fig. 3:**
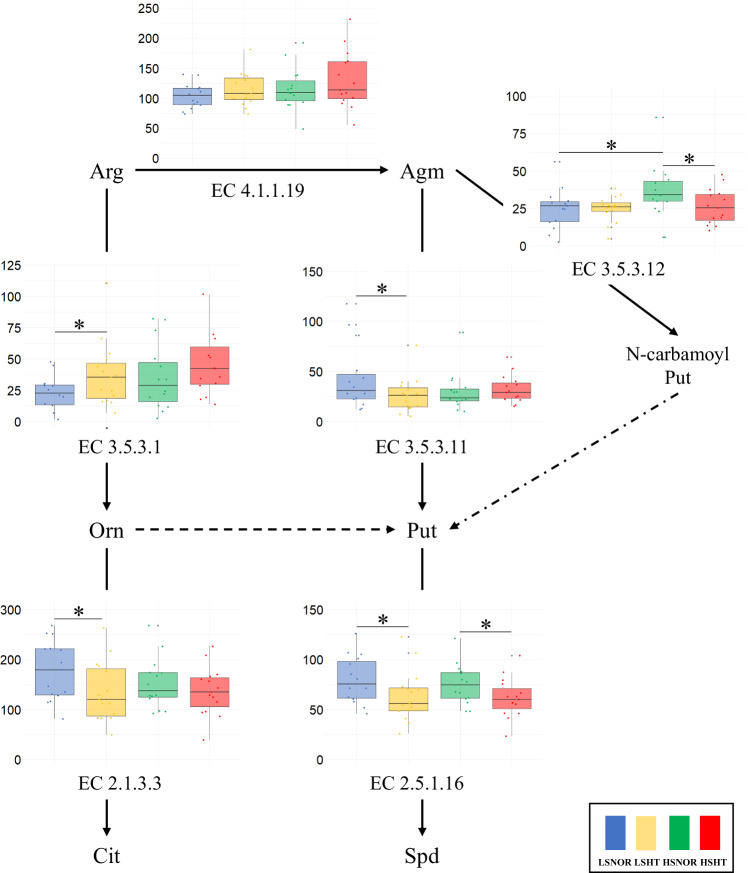
Comparison of gene abundances related to arginine and polyamine metabolism. The gene abundances of arginine- and polyamine metabolism-related enzymes are shown as box plots for each group. The box plot represents the median (central line of the box), first quartile (bottom), and third quartile (top). The whiskers extend to the smallest and largest values within 1.5× the interquartile range (IQR) from the first and third quartiles, respectively. The vertical axis represents the gene abundance in counts per million. Groups with *p* < 0.05 are marked with *. EC.4.1.1.17 and EC.3.5.1.53 accounted for less than 10% of the total, as indicated by the dotted lines. Blue, LSONR; Yellow: LSHT; Green: HSNOR; Red: HSHT, Arg, arginine; Orn, ornithine; Cit, citrulline; Agm, agmatine; Put, putrescine; N-carbamoyl putrescine; SPD, spermidine

Figure 3 shows the gene abundance of arginine- and polyamine metabolism-related enzymes in the four groups. The EC 3.5.3.11, EC 2.5.1.16, and EC 2.1.3.3 values of the LSNOR group were significantly higher than those of the LSHT group (*p* = 0.048, 0.040, and 0.033, respectively), and the EC 3.5.3.1 value of the LSNOR group was significantly lower than that of the LSHT group (*p* = 0.034). The EC 3.5.3.12 and EC 2.5.1.16 values of the HSNOR group were significantly higher than those of the HSHT group (*p* = 0.028 and 0.036, respectively). The EC 3.5.3.12 value of the HSNOR group was significantly higher than that of the LSNOR group (*p* = 0.020). The gene abundances of EC 4.1.1.17 and EC 3.5.1.53 were less than 10% of the total, as shown in Supplementary Fig. S2.

### Enzyme gene abundance and microbial diversity related to arginine–polyamine metabolism

Supplementary Figure S2,A–H illustrates the species-level analyses of enzyme gene abundance. Significant differences in the abundance of arginine- and polyamine metabolism-related enzyme genes (e.g., EC 3.5.3.1, EC 4.1.1.19, and EC 3.5.3.12; *p* < 0.05) were observed in the LSNOR vs. LSHT and HSNOR vs. HSHT group comparisons for several species, especially for *B. vulgatus*, *Eubacterium rectale* (*E. rectale*), and *Roseburia bicirculans* (*R. bicirculans*), as shown in Supplemental Figure S2.

Supplementary Figure S2,A–H (ii, iii) presents the PCA based on bacterial species harboring enzyme genes involved in the arginine–polyamine metabolic pathway. Regarding the α-diversity, significant differences were detected between the LSNOR and LSHT groups for EC 3.5.3.12 (*p* = 0.034) and between the LSNOR and HSNOR groups for EC 2.5.1.16 (*p* = 0.020). In terms of the β-diversity, EC 3.5.3.12 showed a significant difference among the four groups (*p* = 0.027).

## Discussion

The participants were stratified into four groups based on their mean BP and u-NaCl excretion levels. We analyzed the composition of the GM and genes related to arginine–polyamine metabolism. The following three main findings were obtained: First, α-diversity of the GM differed significantly between the LSHT and HSHT groups, as well as between the HSNOR and HSHT groups. Second, the abundance of EC 2.5.1.16, which encodes spermidine synthase with antihypertensive properties, was significantly higher in the normotensive group than in the hypertensive group, irrespective of u-NaCl excretion. Third, significant intergroup differences in bacterial species harboring polyamine metabolic enzyme genes, including EC 2.5.1.16, suggest functional variation in microbial metabolism among the groups.

In contrast to previous reports showing reduced α-diversity in hypertension and salt-sensitive hypertension, our study demonstrated higher α-diversity in the HSHT group [25–28]. Palmu et al. analyzed 6,953 population samples and found significantly lower α-diversity in hypertensive individuals [25]. Kang et al. compared patients with controlled and uncontrolled primary grade 2 hypertension and observed significantly lower α-diversity in the uncontrolled group [26]. Salt intake has also been shown to reduce α-diversity: Cardilli et al. demonstrated that a high-salt diet significantly decreased α-diversity in mice with an intact microbiota [27], while Singh et al. reported in a digital nutrition cohort of 1,014 participants that the Healthy Eating Index, which includes sodium intake as a negative factor, was strongly associated with α-diversity regardless of age or sex [28]. Although these studies suggest that hypertension and salt intake reduce microbial diversity, our results differ in part, likely due to our unique grouping criteria, ethnicity, or age distribution. Nevertheless, both sets of studies agree that α-diversity differs significantly between hypertensive and normotensive individuals.

This discrepancy is likely attributable to differences in participant selection and classification strategy. This classification approach intentionally captured participants located near the centroid of each blood pressure–salt intake category, providing representative samples with average characteristics within each group. However, it may have excluded outlier individuals who could exhibit pronounced salt sensitivity or blood pressure reactivity, which might have contributed to the observed difference in α-diversity patterns compared with earlier studies.

It is well established that human GM can produce polyamines, such as putrescine and spermidine, from arginine [29]. In particular, the arginine decarboxylation pathway (arginine → agmatine → putrescine) is a major route in the human gut, involving arginine decarboxylase (ADC; EC 4.1.1.19) and agmatine ureohydrolase (EC 3.5.3.11). Spermidine synthase (EC 2.5.1.16), which is widely encoded by the bacterial *speE* gene, catalyzes the transfer of a propylamine group from decarboxylated S-adenosylmethionine to putrescine. In our study, the EC 2.5.1.16 gene abundance was significantly higher in the normotensive groups, suggesting that the gut microbial synthesis of spermidine may contribute to BP regulation.

Polyamines are emerging as key modulators in the pathogenesis of [11]. They have been reported to exert cardioprotective, renal, and vascular protective effects, thereby contributing to the reduction of BP [30]. Matsumoto et al. conducted a randomized, double-blind, placebo-controlled parallel trial in 44 overweight adults (BMI 25–30 kg/m²) and found that the intake of yogurt containing *Bifidobacterium animalis* subsp. *lactis* and arginine improved endothelial function, as assessed by endo-peripheral arterial tone. In this group, fecal and serum polyamine concentrations significantly increased [31]. Sieckmann et al. also demonstrated increased expression of polyamine-degrading enzymes in eleven mouse models of kidney injury, including hypertension, suggesting a link between impaired renal function, hypertension, and reduced polyamine levels [32]. Collectively, these findings indicate that polyamines improve cardiovascular and renal function and that a portion of these polyamines are derived from gut bacteria. Therefore, decreased bacterial spermidine synthase expression may contribute causally to HT.

Among various polyamines, spermidine appears to play a particularly key role. In addition to its vasodilatory and anti-atherosclerotic effects, spermidine also suppresses the production of proinflammatory cytokines and improves intestinal barrier function [33, 34]. Increased intestinal permeability facilitates systemic translocation of endotoxins (e.g., Lipopolysaccharide), leading to chronic inflammation and vascular or renal damage, which can exacerbate HT [35, 36]. Spermidine promotes epithelial regeneration and tight junction formation via autophagy induction, strengthening the mucosal barrier and reducing systemic inflammation [33, 37]. In salt-sensitive HT models, high-salt intake disrupts the gut barrier and enhances Th17-mediated mucosal inflammation, thereby elevating the BP [38, 39]. The presence of polyamine-producing bacteria may counteract these effects and maintain intestinal homeostasis.

Although EC 2.5.1.16 expression was associated with BP regulation, its variation alone could not account for the differences associated with salt intake. Moreover, bacteria harboring this gene often possess other metabolic pathways, suggesting that the observed effects on BP may result from complex microbial interactions rather than the function of EC 2.5.1.16 alone.

In this study, we demonstrated that polyamine metabolism in the gut microbiota differed significantly among groups classified by mean blood pressure and urinary salt excretion. However, it is well recognized that the gut microbiota can influence blood pressure through multiple factors, including SCFAs [40, 41], trimethylamine-N-oxide [42, 43], and immune modulation [44]. Future research should focus on integrated comparative analyses that quantify and compare the relative contributions of these factors to identify which pathways play the most pivotal role in blood pressure regulation.

In our dataset, *E. rectale* and *R. inulinivorans*, both harboring EC 2.5.1.16, were more abundant in the HSNOR group than in the LSNOR group, suggesting their contribution to salt resistance in normotensive individuals. Conversely, *R. bicirculans* and *R. intestinalis* were less abundant in the LSHT group than in the HSHT group, potentially indicating that the gut microbial profile is associated with salt sensitivity. These four species share a common functional trait: they produce short-chain fatty acids (SCFAs) and polyamines. *E. rectale* is a major butyrate-producing gram-positive anaerobe in the human gut [45], whereas *R. inulinivorans*, *R. bicirculans*, and *R. intestinalis* belong to the Lachnospiraceae family and ferment dietary fibers into SCFAs, including butyrate and propionate [46–48]. SCFAs have been shown to lower the BP by activating G protein-coupled receptors, reducing vascular resistance, and modulating immune responses [49–51]. Thus, shifts in the abundances of SCFA- and polyamine-producing bacteria may influence the development of salt-sensitive HT.

Moreover, polyamine metabolism in these species may contribute to interspecific metabolic cross-feeding. *R. bicirculans* possesses EC 4.1.1.19, EC 3.5.3.11, and EC 2.5.1.16, allowing it to synthesize spermidine from arginine independently. However, in the gut environment, each step of the polyamine biosynthesis pathway is regulated by distinct enzyme expression patterns across different bacterial species [52, 53]. The availability of arginine and the gut environment, such as pH, also have a significant impact; for example, in *Escherichia coli*, ADC is induced under acidic conditions, leading to the secretion of agmatine [54]. Cross-feeding among gut bacteria can involve a single species that releases agmatine and converts it into putrescine [53, 55]. These cooperative networks may constitute a “hybrid biosynthetic system” that supports sustained polyamine production and contributes to the host physiology.

This study had several limitations. First, this study was a relatively small observational study involving 15 participants per group. Because the subjects were selectively recruited and individuals with extreme blood pressure or sodium intake levels were excluded to minimize bias, the generalizability of the findings is limited, and causal relationships cannot be established. Second, this limitation concerns the collection methods of the analyzed urine and stool samples. Stool samples were obtained only from participants who were able and willing to provide them, and urinary sodium excretion was estimated from a single morning spot urine sample rather than a standardized 24-h collection. Although this approach is practical in a health-screening setting, potential measurement variability and selection bias should be taken into account when interpreting the results. Nevertheless, the cut-off value of 9.7 g/day for urinary NaCl excretion used in this study is consistent with recent national data from Japan. According to the *National Health and Nutrition Surveys* (2017–2024), the median salt intake among Japanese adults has remained stable at approximately 9.7–10.1 g/day over the past decade [56]. Therefore, despite using spot urine instead of 24-h urine collections, this cut-off value was considered a reasonable criterion for stratifying participants in this study. Third, spermidine in humans is also derived from endogenous biosynthesis [11] and dietary intake [10, 11], in addition to the GM [12, 13]; therefore, the relative contribution of microbial synthesis may be limited. Interventional studies, such as fecal microbiota transplantation or bacterial metabolite administration in animal models, are required to validate our findings.

In conclusion, this study characterized the GM composition and polyamine metabolism-related genes based on stratification by BP and salt intake. The increased abundance of EC 2.5.1.16 in normotensive individuals, regardless of salt intake, along with differences in associated bacteria, suggests an important role of gut microbial polyamine synthesis in blood pressure regulation.

These findings underscore the importance of considering gut microbial factors, in addition to host factors, in understanding BP regulation. Despite compositional variation, the GM remains a persistent biological entity that may influence host physiology through metabolic and immune-mediated pathways.

## Supplementary information

Supplemental Figure S1

Supplemental Figure S2

## Data Availability

Raw sequencing data were registered with the DNA Data Bank of Japan (DDBJ; Number DRA018382).
