# Supplementary figures and images for: Gut microbiota–derived polyamine pathways associated with mean blood pressure
Source: Hypertens Res. 2025 Dec 18;49(3):958–68. doi: 10.1038/s41440-025-02490-8 (PMC12960250; doi:10.1038/s41440-025-02490-8)

Supplementary Figure S1

A

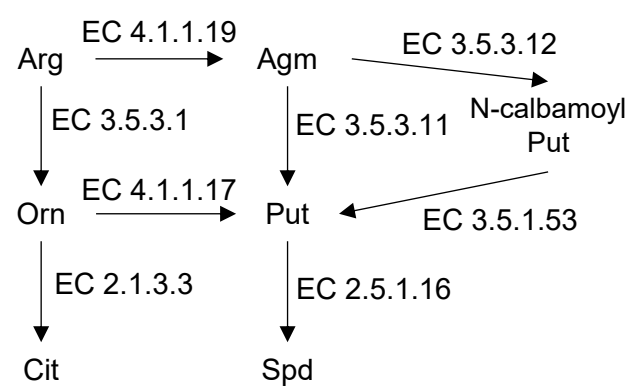

B

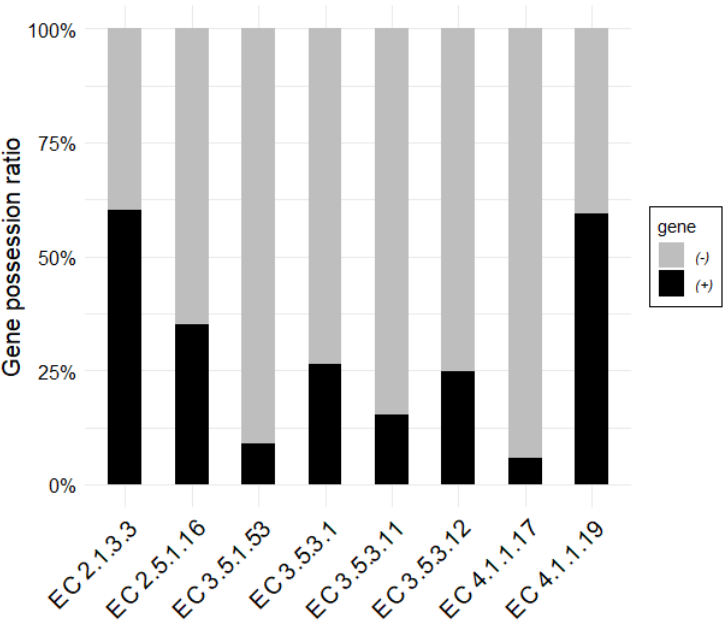

Supplement: Supplementary file 1 — Supplemental Figure S1 [file 41440_2025_2490_MOESM1_ESM.pdf]

# Supplementary Figure S2

A(i)

EC3.5.3.1

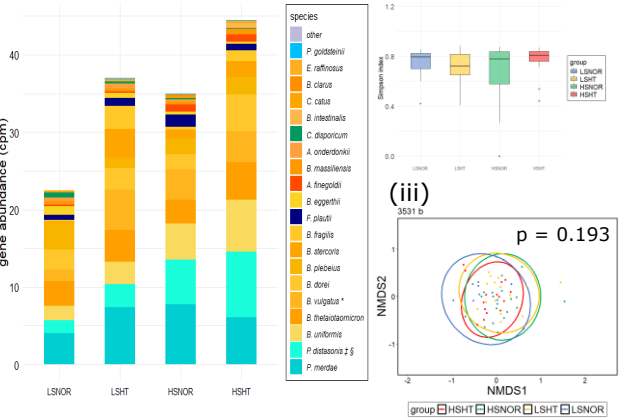

B(i)

EC2.1.3.3

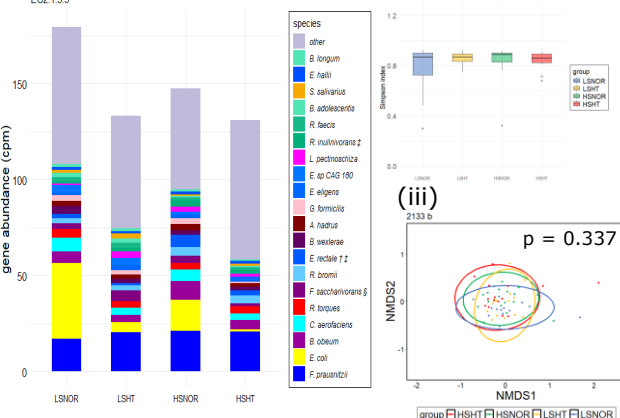

C(i)

EC4.1.1.19

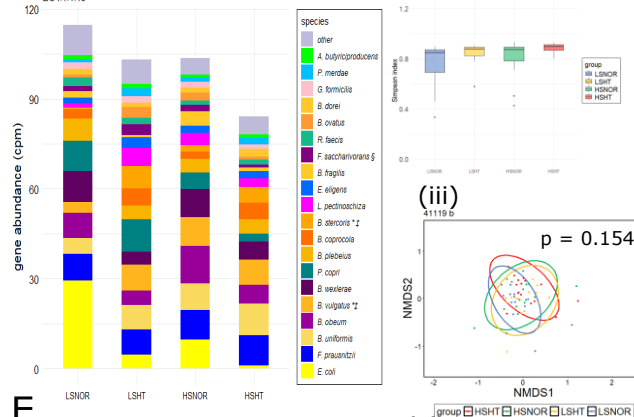

D(i)

EC3.5.3.11

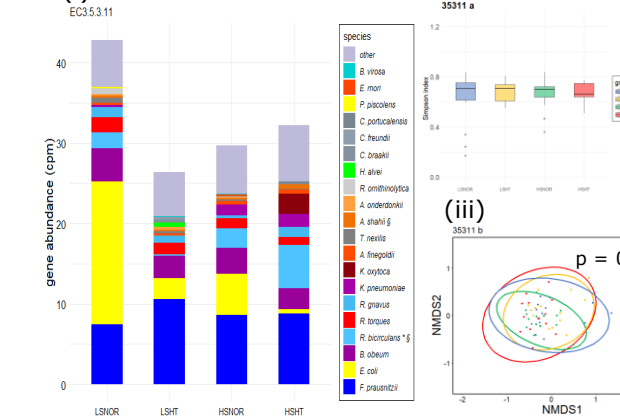

E(i)

EC3.5.3.12

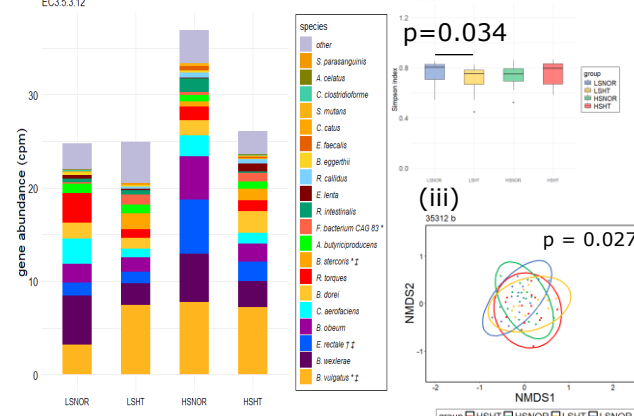

F(i)

EC2.5.1.16

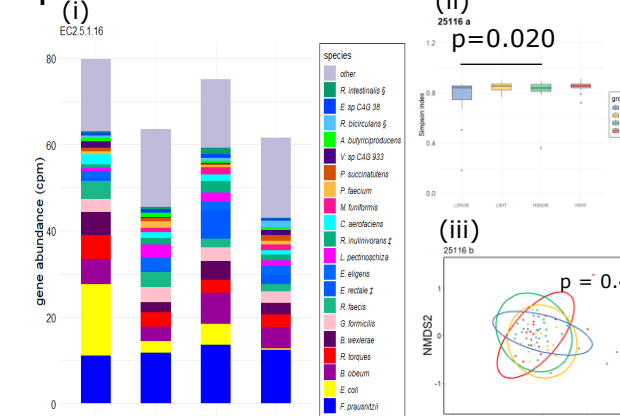

G(i)

EC4.1.1.17

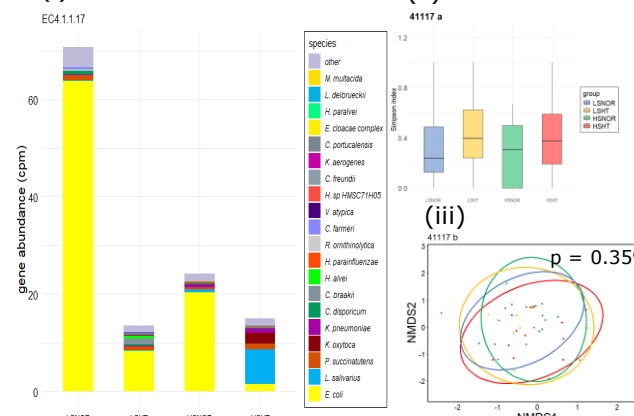

H(i)

EC3.5.1.53

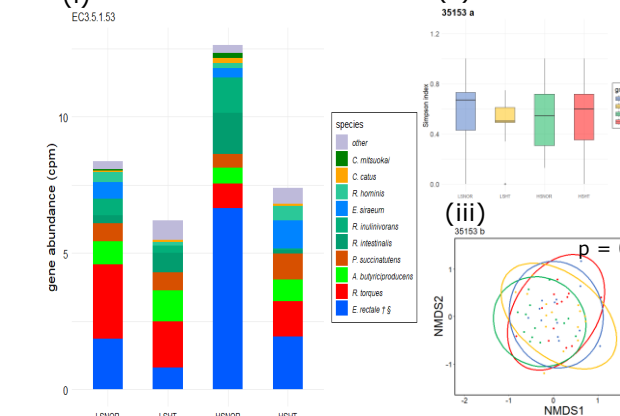

Supplement: Supplementary file 2 — Supplemental Figure S2 [file 41440_2025_2490_MOESM2_ESM.pdf]
